# Underrepresented in medicine students’ perspectives on impactful medical education

**DOI:** 10.1186/s12909-022-03983-7

**Published:** 2022-12-30

**Authors:** Shahrzad Bazargan-Hejazi, Jose A. Negrete Manriquez, Monique McDermoth-Grimes, Elisabeth Alexandra Parra, Deborah Prothrow-Stith

**Affiliations:** 1 College of Medicine, Charles R. Drew University of Medicine and Science, 1731 E. 120th St, Los Angeles, CA 90059 USA; 2 David Geffen School of Medicine at UCLA, 10833 Le Conte Ave, Los Angeles, CA 90095 USA; 3 College of Medicine, Howard University, Washington, DC, USA

**Keywords:** Underrepresented, Medicine, Black, Latino, Medical education, Medical students, Curriculum, Racial minorities, Learning

## Abstract

**Background:**

Exploring the perceptions of underrepresented in medicine (URiM) students about the medical education curriculum and learning environment could optimize their education outcomes. The current study delineated perceptions of URiM medical students about the unique elements and characteristics of an impactful medical education program that create a positive, supportive learning environment culture.

**Methods:**

We conducted in-depth interviews with 15 URiM students between January 2018 and April 2018. Interviewees were recruited from an accredited medical education program in Historically Black Colleges and Universities (HBCUs). The University is also a member of the Hispanic Association of Colleges and Universities in the U.S. The main question that guided the study was, “What do URiM students at a Historically Black Colleges and Universities (HBCU) medical school believe would make a medical education program (MEP) impactful?” We used the grounded theory analytical approach and performed content analysis via qualitative thematic evaluation.

**Results:**

Of 112 enrolled medical students (MS), 15 verbally consented to participation. We identified four general themes and several subthemes. The themes include 1) Grounding learning in the community; 2) Progressive system-based practice competency; 3) Social justice competency and 4) Trauma-informed medical education delivery. Theme 1 included the following subthemes (a) *community engagement*, and (b) *student-run clinic, mobile clinic, and homeless clinic rotations*. Theme 2 includes (a) *interprofessional learning* and (b) *multidisciplinary medicine for cultivating a ‘just’ healthcare system*. Theme 3 includes (a) *longitudinal social justice curriculum*, (b) *advocacy,* and (c) *health disparity research*. Theme 4 had the following subdomains (a) *early and ongoing mentoring* and (b) *provision of supportive policies, services and practices to maximize learning and mental health.*

**Conclusion:**

Our learners found that social justice, trauma-informed, community-based curricula are impactful for URiM learners. These findings highlight the need for further research to assess the impact of permeating the championship culture, community cultural wealth, and transformational education in all aspects of the MEP in providing a supporting and positive learning environment for URiM students.

## Background

The foundation for improving the health and safety of patients starts with training competent medical providers. American Council for Graduate Medical Education (ACGME) identifies Medical Knowledge, Patient Care, Professionalism, Communication, Practice-Based Learning and Improvement, and System-Based Practices as the six core domains that training physicians should know to achieve competence [1]. In 2020, over 20,000 medical students graduated from the U.S medical schools to initiate this process [2]. Additionally, the Liaison Committee on Medical Education (LCME) and the Association of American Medical Colleges (AAMC) acknowledge that diverse populations serve best by a diverse physician workforce [3]. The percentage of matriculated first-year Black or African American students raised by 10.5% in 2020. A similar estimate for Hispanic, Latino or Spanish origin first-year students is reported at 8.6% [4]. However, as a predominately white profession [5], Blacks and Hispanics remain underrepresented in medical schools [6], graduates from medical school, and the physician workforce [6–9].

With the dramatic changes in medical education during the recent year [10], medical students’ perception as the main stakeholder in their learning environment and education outcome has received special attention. The learning environment (i.e., physical structure and services) is strongly associated with the learner’s success, achievements, satisfaction, and wellbeing [11–17]. It is also reported that the learners’ perceptions of the educational curriculum and the learning environment may be swayed by the student characteristics, including racial diversity [18]. The underrepresented in medicine (URiM) students (i.e. students who are a part of racial/ethnic groups that are underrepresented in the physician workforce compared to their numbers in the general population), when compared to their nonwhite counterparts, experience harsher learning environment, impacting their required competencies for graduation [19–21]. Therefore, the URiM students’ perceptions of how to optimize their education climate and outcomes vary from their majority counterparts [3, 21–27].

The URiM students often choose to practice in under-resourced communities hoping to reduce the health care access and equity gap in these communities [18, 28–30]. Yet, empirically we know little about how to better align URiM students learning environment, educational experiences, and institutional support to optimize their success and competency from the perspective of these students [3]. The programs that offer to prepare URiM students, for the most part, reflect the perspective of the medical school and the administration [22, 31–33]. Our study aims to delineate URiM students’ perceptions about any unique elements of an impactful medical education program that create a positive, supportive learning environment culture. Findings from this study could benefit MEPs that aim to nurture URiM students’ educational experiences as learners, educators, researchers, and leaders, enabling them with the skills to serve under-resourced and vulnerable communities.

## Methods

### Design, recruitment, and participants

This qualitative study used grounded theory and non-probability purposive sampling to recruit URiM medical students. *Grounded* theory is a research method that enables the researcher to study distinct experiences by concurrently collecting and analyzing qualitative data from which an explanatory theory could merge [34, 35]. Purposive sampling allows the selection of participants whose insights could be most relevant to the study [35, 36].

The recruitment site was a small inner-city nonprofit university in one of the largest urban underserved areas in the United States. Only 5% of the enrolled students in the university identify as White/Caucasian. Medical students were eligible to participate in this study if they: 1) were 18 and above, 2) were enrolled in the Medical Education Program (MEP), 3) had completed the first year of the program, and 4) identified themselves as Black or African American, or Latinx (Hispanic or Latino), the group we defined as the URiM in this study. Medical students who did not meet these eligibility criteria were excluded from the study. One of the study investigators (M.M.) announced the purpose of the study and its voluntary, anonymous, and confidential nature during different clinical rotations and didactic training sites between January 2018 to April 2018. We used a purposive sampling design [35, 36] and snowball strategy to obtain “information-rich” cases and asked if participants knew individuals with similar characteristics [37, 38]. Students self-identified themselves and contacted the study investigator to schedule the interview.

### Data collection

The 50 to 60-minute one-on-one in-depth interviews were conducted by one of the study investigators (M.M.), who was trained to keep the conversation on track, generate lively and productive dialogue, and obtain balanced input from the interviewees. She first welcomed each participant, introduced the purpose of the interview, and reminded them that negative comments are as helpful as positive comments and that the interview session would be tape-recorded. Participants were assured that information discussed during the interview would remain confidential, and all the subsequent reports would be anonymized. Subsequently, the interviewer responded to all the study-related questions and began the one-on-one in-depth interview.

The main question that guided the study was, “What do URiM students at a Historically Black Colleges and Universities (HBCU) medical school believe would make a medical education program (MEP) impactful?” Probing questions include 1) What elements, resources and/or characteristics would give such a program a competitive advantage and allow it to fulfill its unique mission, i.e., to serve the underserved, under-resourced community; 2) how could that program position itself to best prepare URiM for underserved populations? The study received ethical approval from the university Institutional Review Board (*IRBNet # 1821981–1*).

### Qualitative analysis

Data was collected and analyzed iteratively, as described by Glaser and Strauss [39]. We transcribed tape-recorded conversations verbatim after each interview. We inserted relevant field notes using qualitative analysis software NVivo (Version 12). To conduct thematic analysis and augment data saturation [40], transcripts were read independently by research team members. They looked for common themes and sub-themes within responses for each question. To achieve this, they segmented the data into meaningful analytical units or phrases, marked the segment with descriptive words (coding) and generated a list of the codes (code list) to reapply to new segments of data [41, 42]. Once the initial coding was completed for all segments of the transcribed text, the contents were compared and discussed among the reviewers to achieve consensus regarding to the best themes for organizing the data [41, 42]. Narrow themes were condensed into broader, general ones based on similarities and differences. Then the relationship among general themes and their value in identifying important thematic domains through interview discussions were considered. As recommended in the literature, two independent reviewers outside the study team were assigned to randomly select sections of the transcripts. They followed the process to verify identified codes and themes. Subsequently, coding and thematic issues were discussed, and any disagreement was resolved [37, 43, 44].

### Study creditability

Provisions were made to ensure the triangulation process during data collection and analysis to enhance the credibility of the findings [37, 43, 45]. For example, multiple individuals within and external to the research team, including participants, checked and verified the trustworthiness of the results [46]. They were charged with making sure that all nuances of data were well reflected in the coding and in the selection and categorization of the themes. To guard against researchers’ reflexivity [47], i.e., “researcher’s positionality” (page 678) [48] and ensure the provision of impartial results, the research team members, including the authors in this study, had received training on implicit bias. The implicit bias training informs researchers to be attentive to unconscious thoughts that prompt one to look for findings that fit one’s pre-existing beliefs [48]. Therefore, their feedback and perspectives were considered trustworthy and reliable and enhanced the credibility of data analysis and results. The research team came from different disciplines and had experience conducting qualitative research; therefore, they were highly qualified. The team included two URiM students, a physician, and a medical sociologist.

## Results

Of 112 enrolled medical students (MS), a total of 15 URiM students consented to participation. These individuals participated in a 50 to 60- minutes one-on-one-in-depth interview in April 2018. The student breakdown is as follows: MS2 (*n* = 2), MS3 (*n* = 8), MS4 (*n* = 5). All the participants in the study self-identified as Black or Latinx, and the majority were females, as illustrated in Table 1.

**Table 1:** Participants information (*n* = 15)

Variable	Frequency	Percentage
**Race/Ethnicity**		
° **African American**	**9**	**60.0**
**° Latina**	**6**	**40.0**
**Gender**		
**°** Male	**6**	**40.0**
**° Female**	**9**	**60.0**
**Year in Medical School**		
**° 2nd year**	**2**	**13.3**
**° 3rd year**	**8**	**53.3**
**° 4th year**	**5**	**33.3**

### Emergent themes and subthemes

Our content analysis of URiM students’ perceptions of an impactful MEP yielded four general themes and nine subthemes, as depicted in Table 2.

**Table 2:** Content analysis themes and subthemes

Themes	Subthemes
**1.** Grounding Learning in the Community	***a.*** *Community Engagement* ***b.*** *Student-Run Clinic, Mobile clinic, Homeless Clinic Rotations*
**2.** Progressive System-Based Practice Competency	***a.*** *Interprofessional Learning* ***b.*** *Multidisciplinary Medicine for Cultivating a ‘Just’ Healthcare System*
**3.** Social Justice Competency	***a.*** *Longitudinal Social Justice Curriculum* ***b.*** *Advocacy* ***c.*** *Health Disparity Research*
**4.** Trauma-Informed Medical Education Delivery	***a.*** *Early and Ongoing Mentoring* ***b.*** *Provision of Supportive Policies, Services and Practices to Maximize Learning and Mental Health.*

### Theme 1 (subthemes): grounding learning in the community (community engagement; student-run clinic; Mobile clinic; homeless clinic)

As described in Table 3, the community was noted as an extension of the family and a source of building professional meaning and purpose. Students argued that impactful MEP values build, and strengthen the shared cultural and social capital between URiM students and their community. They described that learning about and engaging with the local community via hot spot mapping activities, clinical rotations, and service-learning activities such as student-run clinics, mobile clinics, and homeless clinics would empower them to build professional competency in the context of the community. (See students’ quotes in Table 3).

**Table 3:** Themes, subthemes, and participants related quotes

**1. GROUNDING LEARNING IN THE COMMUNITY**	
***a. Community Engagement***	
*• “Our community is our family”*	*MS 2, Participant # 6*
*• “As URM students …we desire, to do something in the community, to make a difference, to influence the… decision makers.”*	*MS 4, Participant # 1*
*• “One thing that would be cool to see, that I’ve seen at orientations … is a community walk to nurture our sense of community and passion to serve, which also helps us with mapping out what resources are in the community.”*	*MS 3, Participant # 3*
*• “Some sort of community engagement because I think what separates your average medical student is that constant reminder, desire, to do something in the community, to make a difference.”*	*MS 4, Participant # 2*
***b. Student-Run Clinic, Mobile Clinic, Homeless Clinic Rotations***	
*• “I like the idea of having a possible mobile clinic that is ours because it would really help integrate the community aspect into our curriculum”.*	*MS 3, Participant # 9*
*• “Learning about different medical systems, I don’t know going to Cuba or something to learn about their medical system.”*	*MS 3, Participant # 5*
*• “I think if we’re supposed to be working in teams with other health professionals, …what parts overlap, what can we expect of them in any situation? I think this can easily be done just getting us all together and having a giant overview of what we do”.*	*MS 3, Participant # 10*
**2. PROGRESSIVE SYSTEM-BASED PRACTICE COMPETENCY**	
***a. Interprofessional Learning***	
*• “I think problem-based learning should be focused on team building with interdisciplinary learning, rather than finding the correct diagnosis of a case.”*	*MS 2, Participant # 6*
*• “I think if we’re supposed to be working in teams with other health professionals, …what parts overlap, what can we expect of them in any situation? I think this can easily be done just getting us all together and having a giant overview of what we do.”*	*MS 3, Participant # 10*
*• “Learning about different medical systems,… incorporating that into the curriculum where you could go to a different country, learn about healthcare and then bring positive things ... You know maybe use here especially in the community.”*	*MS 3, Participant # 7*
*• “Learning about different medical systems, I don’t know going to Cuba or something to learn about their medical system.”*	*MS 3, Participant # 5*
***b. Multidisciplinary Medicine for Cultivating a Just Healthcare System***	
*• “…learning how a sociologist would look at our patient population, how a anthropologist, how a psychologist, how they would see this population and our interactions with them, and sort of expand on that wholistic mentality of medicine.”*	*MS4, Participant # 14*
*• “Or bringing in faculty members, like public policy faculty members during the selective or during the doctoring sessions could be away to incorporate those components (*i.e.*, social justice).”*	*MS 3, Participants # 9*
*• “Just a fundamental understanding of how one’s experience directly contributes to their health, whether that be income status or insurance status or whatever the case may be.”*	*MS 3, Participant # 7*
*• “We talk about black people are disproportionately this, or Latinos are disproportionately that, or Asians are disproportionately that. They never talk about what black people have gone through and what those communities of black people ... there’s just so much other things that are affecting these diseases, and we give so much weight and so much clout to the biology, but there’s so much research that’s coming out that’s showing that the social context affects the biology.”*	*MS 3, Participant 12*
**3. SOCIAL JUSTICE COMPETENCY**	
***a. Longitudinal Social Justice Curriculum***	
*• “… the social justice health disparities curriculum within the 1st and 2nd year,..”*	*MS 2 Participants # 8*
*• “As we talk about heart disease or as we talk about kidney function or whatever, talking about things like chronic stress and the research on chronic stress and how that’s related to health inequities. I think there’s so many opportunities across the curriculum to interweave these topics with what we’re learning.”*	*MS 4, Participant # 14*
*• “Whatever can be done to make sure that the curricula on social justice and/or health disparities is required, okay, and I’m emphasizing required because I think it’s easy for people to opt out.”*	*MS 4, Participant # 11*
*• “…actually integrate it into when people are discussing pathophysiology or the physiology of a certain system. I think it’d be a really unique aspect of a program.”*	*MS 2, Participants # 6*
*• “… social justice minded project in the first years of medical school so that we can actually feel like we are contributing to the well-being of our surroundings sooner rather than later.”*	*MS 4 Participant # 4*
***b. Advocacy***	
*• “I would say just more practical advocacy experiences and opportunities.”*	*MS 3, Participant # 5*
*• “I think having us as future physicians be involved with community-based efforts that are not just health access, but talking about the inequities that lead to health disparity. Economic inequities. Things like people being undocumented and what’s the experience like. Things like people being incarcerated. I think having us see ourselves as needing to be a part of that conversation and… not just …provide free blood pressure screenings, but we lend our voices to the concerns of the community.”*	*MS 2, Participants # 8*
*• “I want to learn how to lobby. I want to learn what are the things that I can practically do in these communities from a law perspective, from a criminal justice perspective, or from a health.”*	*MS 3, Participant # 12*
***c. Health Disparity Research***	
*• “This (Senior thesis with health disparity thread) could potentially be the most important thing that you do in medical school.” “…have the opportunity to do some research that’s not just sort of lab-based…”*	*MS 4, Participants # 14*
*• “One thing, I mean, from my perspective is having that ability to have research early on…all the different tools they needed to have to do independent research. And putting that in the beginning and really having a focus on that would really be a great transferable skill ... I think it would increase people’s confidence with doing research.”*	*MS 3, Participants # 7*
**4. TRAUMA-INFORMED MEDICAL EDUCATION DELIVERY**	
***a. Early and Ongoing Mentoring***	
*• “…. I think that mentorship very early should be emphasized, ….*”	*MS 4, Participant # 1*
*• “Introduce mentorship during the 1st year orientation.”*	*MS 2 Participant # 6*
*• “I feel like there’s no such thing as having too many mentors.”*	*MS 3, Participant # 3*
***b. Provision of Supportive Policies, Services and Practices to Maximize Learning and Mental Health***	
*• “I think being able to minimize stress as much as possible is important so I like the idea of pass/fail.”*	*MS 3, Participants # 13*
*• “Moral support during the STEPS, and, preparation for shelf exams.”*	*MS 3 Participant # 12*
*• “Having a financial person who would know aspects of finances that are going to be more prevalent to URM students. A lot of us do not come from a lot of money, who need help financially and need advising when we leave because we don’t necessarily have that wealth accumulated, and wealth knowledge at home.”*	*MS 4, Participants # 14*
*• “…recognizing when students are struggling academically because they’re struggling academically or they’re struggling personally or they’re struggling with family problems, and understanding the difference.”*	*MS 2, Participant # 8*
*• “Let’s not talk so much about the medicine and talk more about just learning. How does a person’s brain work?”*	*MS 3, Participant # 15*
*• “…recognize and place importance on URM students’ mental, physical, spiritual, emotional health.”*	*MS 2, Participant # 6*
*• “And then having mental health providers who look like our students, who understand the interaction between racism, sexism, oppression, and how that’s manifesting in our students’ lives.”*	*MS 3, Participant # 12*

### Theme 2 (the subtheme): progressive system-based practice competency (Interprofessional learning; multidisciplinary medicine for cultivating a ‘just’ healthcare system)

Students believed an impactful MEP would enrich their interprofessional and interdisciplinary experiences. They valued simulation and problem-based learning. They expressed that learning with students from other disciplines such, as nursing and physician’s assistant, builds camaraderie would improve their knowledge and skills in working effectively and efficiently as a team for better patient outcomes. They also expressed that trips, locally and abroad, would build professional competency with the community and nurture a global mindset to better address health disparities. URiM students endorsed learning about medicine beyond the medical model. They suggested that doctoring sessions during the pre-clinical year are ideal to expose students to social science faculty (for example, sociology, anthropology, health policy) in discussion around social context /biopsychosocial approach to disease (Table 3).

### Theme 3 (subthemes): social justice competency (longitudinal social justice curriculum; advocacy; health disparity research)

The general sentiment of the participants was that an impactful MEP would weave social justice competency training into the entire medical education curriculum. As reflected by the students (Table 3), the longitudinal social justice curriculum should begin pre-clinical years to integrate public health issues, racism and other structural prejudice into the discussion of pathophysiology or the physiology of a certain organ system. They further advised it to be a required part of the curriculum. Additionally, students believed that advocacy and health disparity research training would empower them to promote data-informed change (Table 3).

### Theme 4 (subthemes): trauma-informed medical education delivery (early and ongoing mentoring & provision of supportive, policies, services and practices to maximize learning and mental health)

Students expressed the desire to have mentors, especially having the opportunity of having a long-term mentoring relationship. A common belief among students was that mentorship ideally should be started as early as possible, with mentors from various levels of experience, and the relationship should be maintained throughout their medical education. Participants also felt that an impactful MEP would advocates and encompass a trauma-informed approach. For example, provide anxiety prep instead of exam preparedness. They valued having support/professional staff that would understand the multitude of factors that contribute to downgrading academic performance (i.e., financial/familial issues). Additionally, they argued in favor of financial aid services, tuition scholarships and having dedicated time for personal counselling or group discussion on issues that burdens/tax URiM students (for example., racism, sexism, and oppression) (Table 3).

## Discussion

Our qualitative study highlights four general themes reflecting URiM students’ perceptions regarding the impactful components of an MEP.

### 1^st^. Grounding learning in the community

By virtue of being HBCU students, our participants expressed that an impactful MEP is poised to ground the entire curriculum in a community-driven mission with familial feelings. They believed these values could be realized by exposing students to diverse community-based rotation sites for exposure to different vulnerable patient populations. The concept of community cultural wealth [49] affirms that once learning is grounded in the community with familial culture, it supplies students with deeper insight into the clinical concern, diagnosis and clinical management [50]. Additionally, community-engaged service-learning pedagogy nurtures students’ social responsibility attitude and commitment to serve and mitigate health disparities of the underserved and under-resourced communities. It is suggested that a medical education cognizant of “community as teachers” [51] is more likely to graduate physicians who behave ethically and professionally and deliver culturally effective care [51–53].

### 2^nd^. Progressive system-based practice competency

Our findings reveal that the multidisciplinary and interprofessional learning approach is impactful because it teaches the language of the local and global healthcare system [5, 54–57]. A view that supports the goals of the Liaison Committee on Medical Education (LCME) standards [58]. Multidisciplinary and interprofessional collaboration build on the cooperative practice between a team of providers and a patient, leading to a decision that is informed by the patient’s social determinants of health [59, 60]. As reported, medicine is a team service profession where team members need to know their role in different disciplines [61] to refine their coordinated-care management skills and the delivery of safe and quality patient care [62–64]. Indeed, the existing experiential education in MEPs provides the opportunity for students from different disciplines to interact, collaborate and coordinate care [59]. .However, by virtue of their diversity and intrinsic social responsibility, to deliver equitable services, our medical students argued that the system-based practice teaching should be fused with conversation around the political, institutional, and social aspects of the health and health care delivery system. A conversation that falls short in” professionalism” and “system-based practice” competencies [65] and could plant seeds for questioning the current approach to system-based practice in healthcare and encourage a progressive version of this competency [66].

### 3^rd^. Social justice competency

According to our findings, social justice curriculum in a medical education program would increase its impactful utility by nurturing the URiM students’ social-justice attitude and skills to remedy sources of health disparity. These findings echo others who believe in incorporating structural competency in the medical education curriculum to prepare future medical providers to address injustices in healthcare delivery systems [65, 67–73]. Proponents of this approach believe that physicians should be trained to understand the structural and social determinants of health through a theoretical lens to eradicate health inequality [57, 65, 74]. Others suggest that the structural competency rotations have successfully linked theory with practice, making it possible for the students/residents to work with communities-based organizations to actively reshape the inequitable healthcare landscape [57, 75, 76].

Additionally, the URiM students’ endorsement of health disparity research underlines their commitment to empirically validate the structural and social factors that determine the health of the underserved, under-resourced and vulnerable populations. The notion of creating a culture of inquiry by conducting research during medical education is reflected in the number of institutions providing protected time for the students to conduct independent or mentor-guided research (i.e., 65 medical schools in 2017–2018) [77, 78] Others contend that medical education that provides research training advances their students’ professional pathway, as well as the “health of the patients and communities they serve.” [10, 79, 80] Physician-scientists are better positioned to intervene within their setting, leveraging the evidence for advocacy and demonstrating structural competency [73, 75, 81].

### 4^th^. Trauma-informed program delivery

Our findings suggest that an impactful MEP would be committed to thriving URiM students by providing an ongoing culturally congruent mentorship and implementing supportive policies and practices. Mentorship has increased medical students’ career satisfaction, retention, research productivity, self-efficacy, and career development [6, 82, 83]. Establishing early and ongoing relationships with mentors with similar strengths, experiences, and cultural wealth mitigates the stress and anxiety associated with being the first in the family to finish college and the apprehension of a high-stress learning environment [3, 83–85].

Moreover, as reflected in our findings, others have validated the importance of supportive policies, services, and practices in the MEP [86] to buffer minority students’ additional stressors such as the minority tax [87], hidden curriculum [88], impostor syndrome [89], and mental health stressors [26, 90]. .As a result, our findings suggest that a genuinely impactful medical education program would be cognizant of these barriers and root causes and consider the trauma-informed MEP program to thrive for its students [26, 86].

### Limitations

Our research approach has limitations, including a lack of generalizability to other URiM students in other medical schools. Still, it offers a range of insights which would be challenging to explore based on quantitative analysis. For example, amid systemic racism’s deleterious effects on URiM students and their future career goals [28], our approach allowed participants to focus on uplifting insights, reflections, and experiences that are not often told [91]. Furthermore, our study builds problem identification to describe solutions. For example, the URiM students view curricula on social justice as an essential component of medical education and provide solutions to mitigate the effects of racism in medical education.

Nonetheless, the methods used to collect data in the study allow for possible confirmation bias or social desirability. However, to minimize this limitation, the medical student interviewer was trained to balance the power dynamic, stay task-oriented, and obtain well-rounded input [92]. Additionally, to safeguard against Hawthorn or observer effect (i.e., a threat to internal validity), the interviewer assured participants of the anonymity and confidentiality of their participation and responses in the final report.

## Conclusions

In summary, the URiM students perceived an MEP as impactful when it 1) integrates learning with community engagement; 2) targets progressive system-based practice competency; 3) builds social justice competency, and 4) delivers trauma-informed medical education. Our findings reinforce the significance of understanding the values of cultivating championship culture [93], investing in community cultural wealth [49], and aiming for a transformational MEP that is aligned with the URiM students’ goal of serving and advocating for their communities while maximizing their mental health and minimizing the minority tax. Cultivating a championship culture could help students build a strong and meaningful sense of belonging and relationship with their peers, staff, and faculty, building trust and transparency in all aspects of their education and performance. Additionally, an MEP that approaches students of color from the community cultural wealth lens, i.e., students who possess cultural capital, could better relate and communicate with them to transform the learning environment and maximize their professional and personal growth potentials. In light of the current health, economic, and racial injustice pandemics, the MEPs should invest in identifying strategies and resources needed to actualize values that support and provide a positive learning environment for URiM students. Our findings could shed some light on that direction and guide decision-makers in improving their curriculum. Further research is needed to address these issues with a larger number of URiM students. Additionally, research is required to assess the impact of permeating the championship culture, community cultural wealth, and transformational education in all aspects of the MEP in providing a supportive and positive learning environment for URiM students.

## Data Availability

All data generated or analyzed during this study are fully available without restriction and from the time of publication. The data supporting this study’s findings are available on request from the corresponding author (SHB). The data are not publicly available because they contain information that could compromise the privacy of research participants.
